# The Effect of Mechanochemical Treatment of the Cellulose on Characteristics of Nanocellulose Films

**DOI:** 10.1186/s11671-016-1632-1

**Published:** 2016-09-20

**Authors:** V. A. Barbash, O. V. Yaschenko, S. V. Alushkin, A. S. Kondratyuk, O. Y. Posudievsky, V. G. Koshechko

**Affiliations:** 1 National Technical University of Ukraine “Kyiv Polytechnic Institute”, Prospect Peremogy 37, Kyiv, 03056 Ukraine; 2 L.V. Pisarzhevsky Institute of Physical Chemistry of the National Academy of Sciences of Ukraine, Prospect Nauki 31, Kyiv, 03028 Ukraine

**Keywords:** Mechanochemical treatment, Hydrolysis, Nanocellulose film, Transparency, Young’s modulus, Tensile strength

## Abstract

The development of the nanomaterials with the advanced functional characteristics is a challenging task because of the growing demand in the market of the optoelectronic devices, biodegradable plastics, and materials for energy saving and energy storage. Nanocellulose is comprised of the nanosized cellulose particles, properties of which depend on characteristics of plant raw materials as well as methods of nanocellulose preparation. In this study, the effect of the mechanochemical treatment of bleached softwood sulfate pulp on the optical and mechanical properties of nanocellulose films was assessed. It was established that the method of the subsequent grinding, acid hydrolysis and ultrasound treatment of cellulose generated films with the significant transparency in the visible spectral range (up to 78 % at 600 nm), high Young’s modulus (up to 8.8 GPa), and tensile strength (up to 88 MPa) with increased ordering of the packing of the cellulose macromolecules. Morphological characterization was done using the dynamic light scattering (DLS) analyzer and transmission electron microscopy (TEM). The nanocellulose particles had an average diameter of 15–30 nm and a high aspect ratio in the range 120–150. The crystallinity was increased with successive treatments as shown by the X-ray diffraction (XRD) and Fourier transform infrared spectroscopy (FTIR) analysis. The thermal degradation behavior of cellulose samples was explored by thermal gravimetric analysis (TGA).

## Background

The development of the nanomaterials with improved functional characteristics is a challenging task at present because of the growing demand in the fields of optoelectronics, materials for energy saving and storage [1–5]. Nanocellulose is a group of nanomaterials that consists of the nanosized cellulose particles. Characteristics of nanocellulose particles depend on properties of plant raw materials and methods used in the production [6, 7]. Nanocellulose that is produced from the renewable lignocellulose materials has improved mechanical properties, such as high surface area-to-volume ratio and high aspect ratio [8–10]. Nanocellulose often replaces well-known material such as glass and certain polymers, which are not biodegradable at ambient conditions, in order to create new specific nanocomposites, adsorbents, and functional materials for the electrodes in the chemical sources of power and optoelectronic devices [11–16], biodegradable plastics and paper with special characteristics [17–19].

Cellulose is one of the most abundant biopolymers in our planet with the annual production of up to 10^11^ tons [6]. During the process of photosynthesis, cellulose macromolecules form nano- and microfibril structures stabilized by the hydrogen bonds [7]. Nanocrystalline cellulose, which consists of rod-shaped crystals with 2–20 nm cross-section diameter of and length from 100 nm to several micrometers [17], is prepared by removing amorphous regions from the cellulose. Nanocrystalline cellulose can form stable aqueous suspensions with chiral nematic properties and, as cholesteric liquid crystals, retains optical properties in films even after solvent evaporation [18]. Nanocrystalline cellulose is characterized by the strength five times higher than that of steel and a coefficient of thermal expansion of less than that of quartz [19]. The network formation of nanosized particles in nanocrystalline cellulose films determines its optical transparency. In this network, the diameter of these particles is much smaller than the light wavelength, which significantly decreases light scattering as compared to the regular fibers [20].

Nanocellulose is prepared by mechanical, chemical, and enzymatic methods. Mechanical methods employ various forces to reduce the size of the natural cellulose fibers to nanoscale. This approach includes multiple passages of the cellulose fibers through a high-pressure homogenizer and leads to the significant energy consumption (above 25 kW/kg) [6]. Spence et al. [21] have shown that the homogenization process is the most expensive method for the nanomaterial isolation. Chemical methods are based on the cleavage of 1–4 glycosidic bonds of the cellulose chains and an isolation of the cellulose nanocrystals eliminating the amorphous cellulose part [22]. Enzymatic methods generate nanocellulose through the biosynthesis from monosaccharides or fermentation of the cellulose fibers. The enzymatic methods are time-consuming and require expensive reagents. In some instances, the initial enzymatic treatment of the cellulose prior to the mechanical grinding can decrease the energy consumption required for the preparation of nanocellulose [23]. For these reasons, a pre-treatment of the fibrous material is usually performed in order to decrease the size of the cellulose fibers and to ease the fibrillation and the process of nanocellulose preparation.

In the present study, the effects of the treatment of the bleached softwood sulfate pulp to nanocellulose followed by mechanochemical treatment on the optical (transparency in the visible spectrum range) and mechanical properties (Young’s modulus, tensile strength) of the nanocellulose films were investigated. The grinding process using the standard for pulp industry equipment was used as a pre-treatment of the cellulose to decrease the size of the fibers and to reduce acid consumption in the process of the hydrolysis. Additionally, in comparison to the high-pressure homogenization processes, it does not have the blocking problems generated by larger fibers [24]. We used the bleached softwood sulfate pulp as a starting material since it is highly produced by the paper industry.

## Methods

Mechanical treatment of the bleached softwood sulfate pulp (Arkhangelsk CPF, Russia) was performed using laboratory grinding complex LRK-1 (UkrSRIP, Ukraine) with the setting of diamond garniture from 0.1 to 0.4 mm for reaching 93 Schopper-Riegler (SR) degrees freeness. Measurements of the beating rate were carried out by SR-2 device (CSRIP, Russia). The pulp contained approximately 92.3 % cellulose, 5.7 % hemicelluloses, 0.23 % lignin, and 0.21 % ash. The chemical composition of the pulp was determined by the methods described by TAPPI standards [25].

Hydrolysis of the grinded cellulose was carried out by sulfuric acid solutions with different concentration (from 18 to 64 %) at the liquid-to-solid ratio of 44:1 during 5–60 min. The calculated amount of sulfuric acid with the corresponding concentration was slowly added into the flask with the cellulose suspension, and the required volume of the acid with concentration above 50 % was added drop-wise. The temperature of the reaction was maintained in the range from 20 ± 1 to 60 ± 3 °C. Upon expiration of the reaction time, the hydrolysis was stopped by tenfold dilution with distilled water and cooling of the suspension to the room temperature.

The hydrolyzed cellulose was washed three times by the centrifugation (8000 rev/min) and subsequent dialysis until reaching neutral pH. Ultrasound treatment was performed using ultrasound disintegrator UZDN-A (SELMI, Ukraine) for 5–60 min. The cellulose dispersion was placed in an ice bath to prevent overheating during treatment. Eventually, the suspension had taken the form of a homogenous gel-like dispersion.

The prepared dispersions were poured into Petri dishes and dried at room temperature in air to obtain cellulose films. Their density was determined according to the ISO 534:1988. The degree of polymerization (DP) was determined by the viscosity of the samples dissolved in copper ethylene-diamine solution according to ISO 5351.

The determination of particle diameter’s distribution for cellulose dispersions was performed by dynamic light scattering (DLS) using analyzer Zetasizer Nano (Malvern Instruments, UK). Transmission electron microscopy (TEM) images were obtained using electron microscope TEM125K (SELMI, Ukraine) operating at a potential of 100 kV. A delute suspension (0.1 wt.%) was dropped onto a thin scaffoldings Lacey Formvar/Carbon, 400 mesh, copper approx. grid hole size 42 μm (TED PELLA, Inc, USA). Electron absorption spectra of the nanocellulose films in UV, visible and near infrared regions were registered on two-beam spectrophotometer 4802 (UNICO, USA) with resolution of 1 nm. Fourier transform infrared spectroscopy (FTIR) spectra were measured using spectrophotometer IFS66 (Bruker, USA) with resolution of 2 cm^−1^. X-ray diffraction patterns of the different cellulose samples were obtained by Ultima IV diffractometer (Rigaku, Japan). The method proposed in [26] was used to determine the crystallinity degree (CD) of the samples, in terms of which CD = (*I*_200_ − *I*_am_)/*I*_200_ × 100 %, where *I*_200_ is an intensity of (200) reflex about 23°, *I*_am_ intensity of amorphous scattering at 18.5°.

Tensile properties of the nanocellulose films were measured at controlled temperature (23 ± 1 °C) and humidity (50 ± 2 %) according to ISO 527-1. Tension tests were performed at a crosshead speed of 0.5 mm/min on the TIRAtest-2151 (Germany) instrument equipment with a 2N load stress. For testing, test strips with 10 ± 2-mm wide and 25 ± 5-mm long were used. The data reported are tensile strength and Young’s modulus. Each composition was tested with a minimum of five specimens to extract an average and standard deviation for each property.

The thermal degradation behavior of cellulose samples was explored by heating using Netzsch STA-409 thermoanalyzer. The samples were heated at a rate of 5 °C/min, from 25 to 450 °C.

## Results and Discussion

**Fig. 1:**
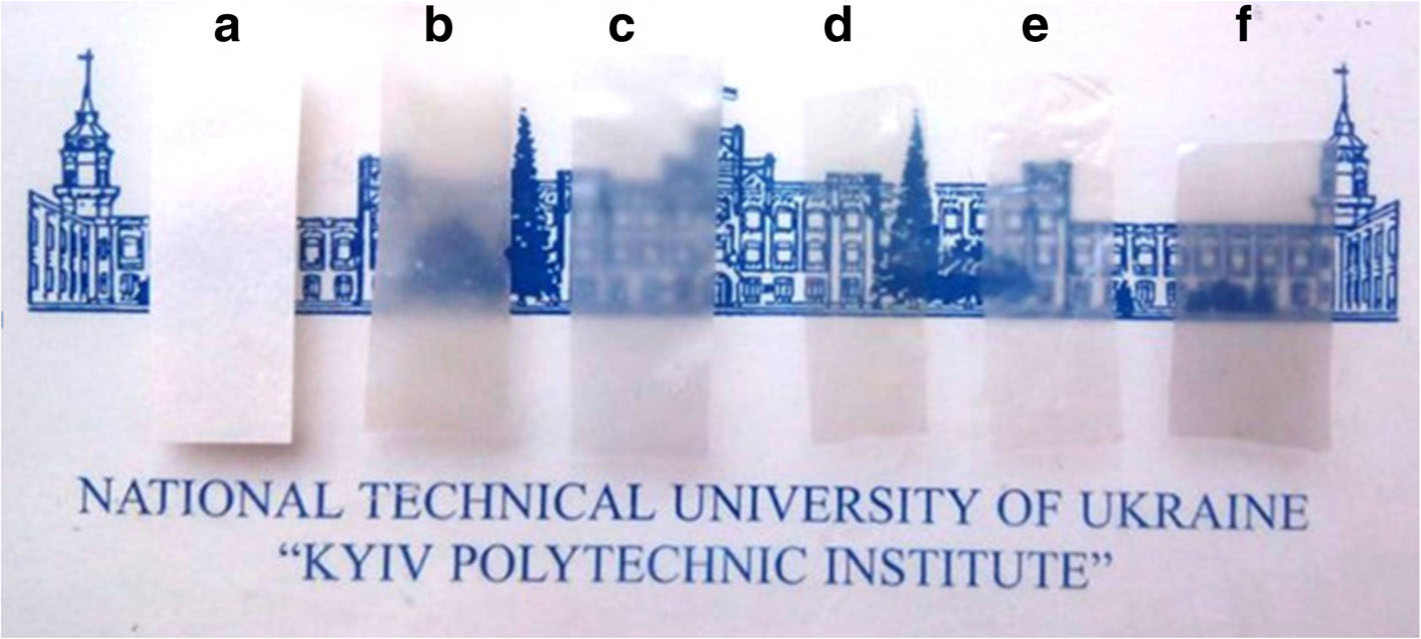
Transparent sheets prepared from cellulose dispersion after grinding (**a**), after grinding and hydrolysis by sulfuric acid of concentration 18 % (**b**), 30 % (**c**), 43 % (**d**), 50 % (**e**), and 64 % (**f**) with ultrasound treatment

**Fig. 2:**
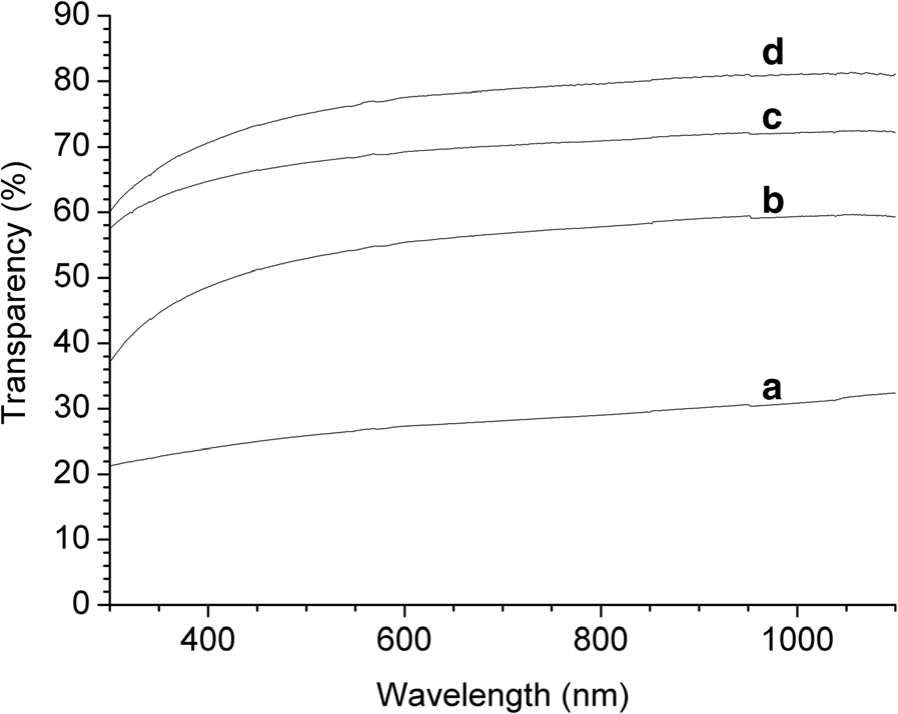
Electron absorption spectra of the cellulose films prepared from cellulose dispersion after grinding and hydrolysis by sulfuric acid of concentration 30 and 43 % without (**a**, **b**) and with ultrasound treatment (**c**, **d**), respectively

First, we assessed the properties of the cellulose that was used in the preparation of nanocellulose. The spectral analysis demonstrated that the samples of non-hydrolyzed cellulose were non-transparent in the visible spectral range (Fig. 1a). The acid hydrolysis and the ultrasound disintegration of the cellulose suspensions prepared by the mechanochemical treatment have led to the increased stability of the aqueous dispersions and generation of homogeneous gel, which did not sediment, resulting in the formation of the transparent cellulose films (Fig. 1b–f). The electron absorption spectra of the cellulose films prepared from different dispersions after acid hydrolysis of the grinded cellulose with and without subsequent sonication demonstrated that the hydrolysis of cellulose leads to the formation of films with a transparency of more than 50 % in the visible spectral range (Fig. 2). Additionally, we observed a significant increase of the transparency of cellulose films treated with the sonication. The transparency of the film prepared by the hydrolysis of the cellulose with 43 % sulfuric acid and ultrasound treatment was 78 % at the wavelength of 600 nm (Fig. 2). The value of transparency of the films prepared in this study is higher (relative transmittance) than the transparency of the cellulose nanofiber films obtained from bleached kraft eucalyptus, acacia, and pine pulps reported previously (40–65 %) [27].

**Fig. 3:**
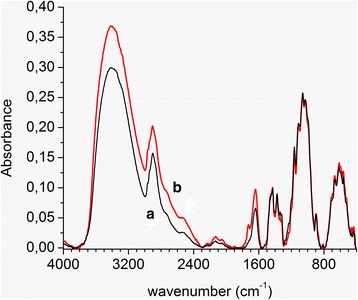
FTIR spectra of the initial cellulose (**a**) and cellulose hydrolyzed at concentration of sulfuric acid 64 % (**b**)

We also observed that the hydrolysis of the cellulose at high concentrations of sulfuric acid (above 50 %) led to the decrease in the transparency of films which acquired brownish color due to the destruction of the cellulose macromolecules which was confirmed by the FTIR analysis (Fig. 3). The band around 1730 cm^−1^ was detected in the spectrum of the hydrolyzed sample indicating the presence of the carbonyl groups in the macromolecules, which is the characteristic of the chromophore groups.

The increase in the transparency of the cellulose films suggests that the mechanochemical treatment followed by the sonication procedure results in the decrease of the hydrolyzed cellulose particle size through more dispersivity of nanocellulose. The decrease of the cellulose particle size and the increase of its dispersivity were assessed by measuring the changes in the degree of polymerization (DP). Thus, DP of the initial pulp was 1037; DP pulp grinded in laboratory grinding complex up to 93 °SR was 635; DP after hydrolysis with 43 % sulfuric acid was 305; DP after additional ultrasound treatment was 110; and DP after hydrolysis with 64 % sulfuric acid was 53. The decrease in the DP confirmed the degradation of the cellulose during the mechanochemical treatment.

**Fig. 4:**
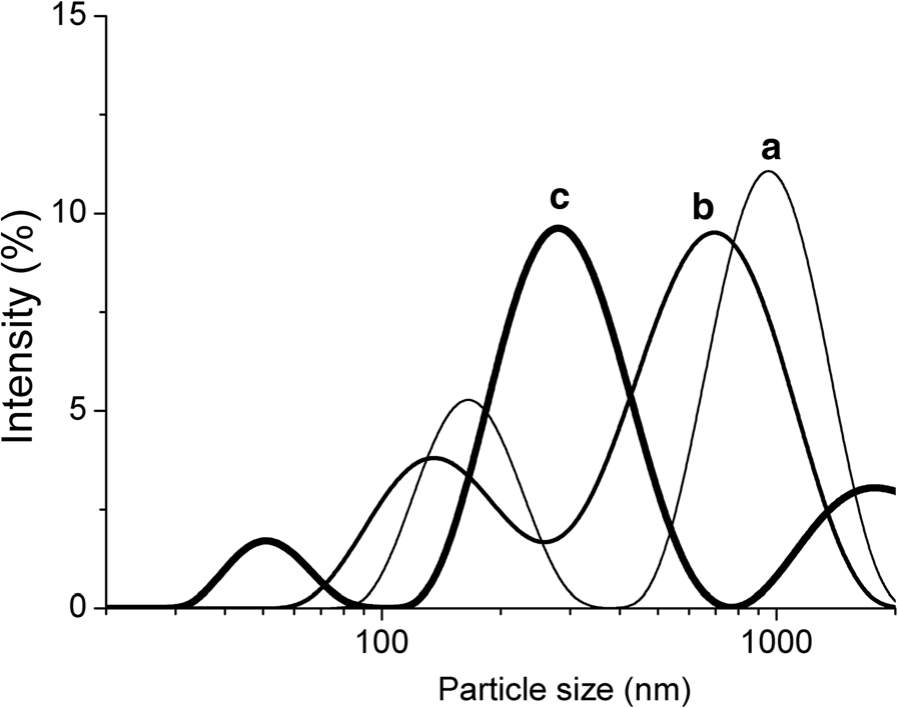
Nanoparticles size distribution for dispersions prepared by ultrasound disintegration of the cellulose samples hydrolyzed by sulfuric acid of different concentration: 30 % (**a**), 43 % (**b**), and 50 % (**c**)

To further assess the changes in the particle size induced by hydrolysis and sonication, we performed the dynamic light scattering analysis and TEM. The dynamic light scattering analysis demonstrated that the nanocellulose suspensions had polydisperse distribution of particle diameters, which should facilitate the formation of more dense and more transparent cellulose films (Fig. 4). It suggests that the hydrolysis by sulfuric acid of higher concentration leads to the formation of the smaller nanoparticles, which is consistent with the DP data.

**Fig. 5:**
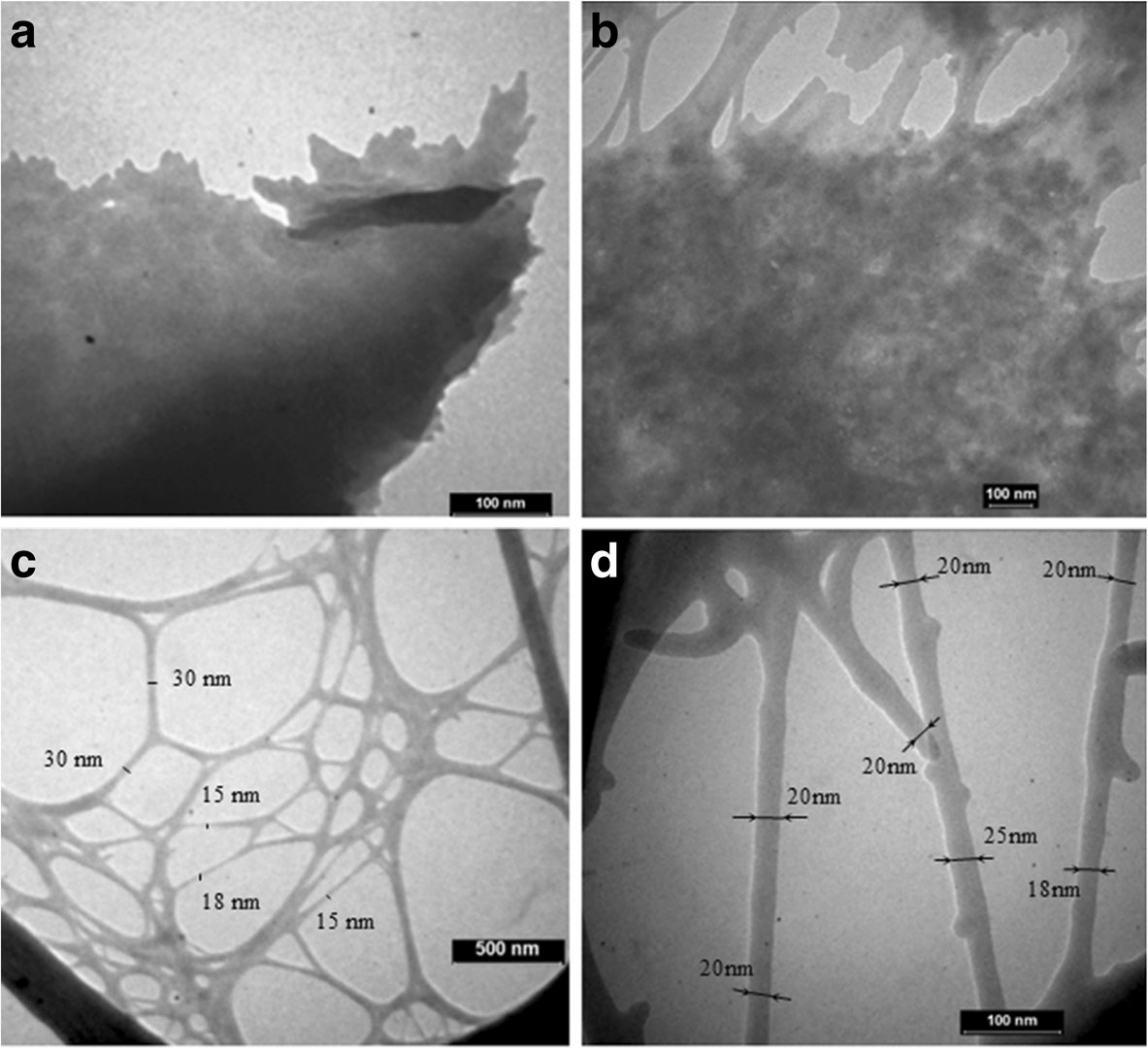
TEM images of the nanocellulose samples after different conditions of hydrolysis with sulfuric acid and ultrasound treatment for 30 min: 30 % at 20 °C, 30 min reaction time (**a**); 64 % at 20 °C, 5 min reaction time (**b**); 43 % at 45 °C, 45 min reaction time (**c**, **d**)

Next, the morphology of the nanocellulose samples using TEM was examined. The mechanochemical treatment of the cellulose leads to the formation of different networks of nanocellulose depending on the conditions: concentration of acid, temperature, time of the hydrolysis, and ultrasound treatment (Fig. 5). The analysis of the TEM micrographs revealed that the aqueous suspension of nanocellulose consist of rodlike nanoparticles, whereby there were some nanoparticles agglomerated in the forms of bundles while some of them were well separated. The agglomeration of separate nanoparticles led to the formation of a dense layered structure (Fig. 5a, b). The formation of agglomerates was likely due to the strong interaction between the particles induced by chemical modifications of the cellulose macromolecules during acid hydrolysis (Fig. 3) and due to the Van der Waals attraction forces between nanoparticles. The cellular networks (Fig. 5c) were formed from the nanoparticles with the diameter in the range of 15–30 nm (Fig. 5d) and aspect ratio in the range of 100–150. The morphology of nanocellulose obtained in this study was like nanocellulose produced by an acid hydrolysis according to the results obtained by authors [28].

The mechanical properties of the nanocellulose films (Table 1) were determined. An increase of the acid concentration used in hydrolysis from 18 to 43 % has led to an increase of the mechanical properties of the nanocellulose films: Young’s modulus up to 8.8 GPa and tensile strength up to 88 MPa. publications have reported the tensile properties of cellulose nanofiber films. Zimmerman et al. [29] reported Young’s modulus values of nanofibrillated cellulose films in the range of 5.5 to 6.0 GPa. In a separate study [30], Young’s modulus values of films generated from nanofibrillated bleached pulp, wheat straw, and recycled newspaper were between 6 and 9 GPa.

**Table 1:** The effect of sulfuric acid concentration, temperature, duration of hydrolysis, and ultrasound treatment on density and mechanical properties of the nanocellulose films

Concentration H_2_SO_4_, %	Temperature, °C	Duration of hydrolysis, min	Duration of the ultrasound treatment, min	Density, g/cm^3^	Tensile strength, MPa	Young’s modulus, GPa
18	60	50	5	1.01 ± 0.04	26.1 ± 1.4	2.1 ± 0.18
	60	50	30	1.03 ± 0.02	28.0 ± 2.2	2.5 ± 0.11
	60	50	60	1.14 ± 0.05	31.9 ± 1.7	3.1 ± 0.12
	60	50	90	1.27 ± 0.03	38.7 ± 1.6	3.7 ± 0.14
30	20	30	60	1.13 ± 0.03	61.2 ± 3.9	6.3 ± 0.33
	20	90	60	1.16 ± 0.06	63.1 ± 3.6	5.9 ± 0.29
	60	30	60	1.23 ± 0.04	63.5 ± 2.4	5.7 ± 0.14
	60	50	60	1.25 ± 0.03	69.0 ± 5.0	5.5 ± 0.31
	60	90	60	1.26 ± 0.02	72.8 ± 4.3	5.7 ± 0.19
43	60	5	30	1.56 ± 0.05	88.0 ± 7.4	8.8 ± 0.16
	60	5	60	1.43 ± 0.04	77.2 ± 6.3	7.3 ± 0.25
	60	10	30	1.51 ± 0.04	84.1 ± 5.3	7.8 ± 0.37
	60	15	30	1.45 ± 0.02	78.3 ± 4.6	7.1 ± 0.48
	60	20	30	1.41 ± 0.03	74.3 ± 5.8	6.7 ± 0.35
50	20	60	60	1.34 ± 0.04	60.3 ± 4.8	5.5 ± 0.36
	60	30	60	1.31 ± 0.05	64.5 ± 4.2	5.2 ± 0.45
	60	90	60	1.33 ± 0.03	66.7 ± 4.1	5.1 ± 0.33
	70	5	5	1.38 ± 0.04	73.8 ± 3.4	6.2 ± 0.41
64	20	30	30	1.11 ± 0.03	50.0 ± 4.4	4.4 ± 0.38
	20	30	60	1.12 ± 0.03	51.3 ± 5.3	4.6 ± 0.32
	45	30	30	1.42 ± 0.06	56.7 ± 5.6	4.5 ± 0.29
	45	45	30	1.36 ± 0.03	67.4 ± 3.6	6.8 ± 0.35
	60	10	30	1.45 ± 0.04	83.8 ± 6.4	7.3 ± 0.43

Further increase of the acid concentration (above 50 %) used in the hydrolysis leads to a sharp decrease of all strength properties. The hydrolysis with low concentration of sulfuric acid affected predominantly amorphous regions of the cellulose, compared to crystalline ones, and led to an increase of the mechanical properties of the films. The hydrolysis with high concentrations of sulfuric acid (50–64 %) affected both amorphous and crystalline regions of the cellulose and led to the formation of films with the brownish color mentioned above.

**Fig. 6:**
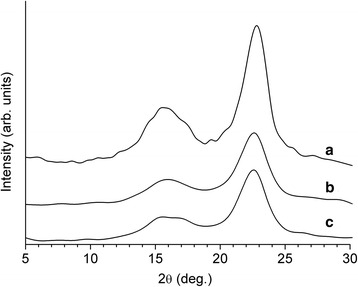
X-ray diffraction patterns of the cellulose samples: initial cellulose (**a**), cellulose after grinding and 43 % sulfuric acid hydrolysis (**b**), and cellulose after additional sonication (**c**)

Cellulose samples were examined by X-ray diffraction to confirm these findings. The X-ray diffraction patterns revealed that grinding, hydrolysis, and ultrasound treatment affected cellulose crystallinity (Fig. 6). The crystallinity degree of the initial cellulose, the hydrolyzed cellulose, and the sonicated cellulose was 75, 78.3, and 79.8 %, respectively. These data indicate that these treatments increased the package ordering of the macromolecules due to the decrease in the ratio of amorphous parts of cellulose.

Next, the cellulose samples were analyzed by FTIR. Previously published data [31] suggest that the degree of crystallinity of cellulose changes symbatically to the ratio between the intensity of the bands at 1430 and 900 cm^−1^ (Fig. 3). In our dataset, the ratio between the intensity of the bands at 1430 and 900 cm^−1^ for the initial cellulose, the hydrolyzed cellulose, and the sonicated cellulose was 1.74, 1.82, and 1.86, respectively. These data correlate well with the data from X-ray diffraction described above.

**Fig. 7:**
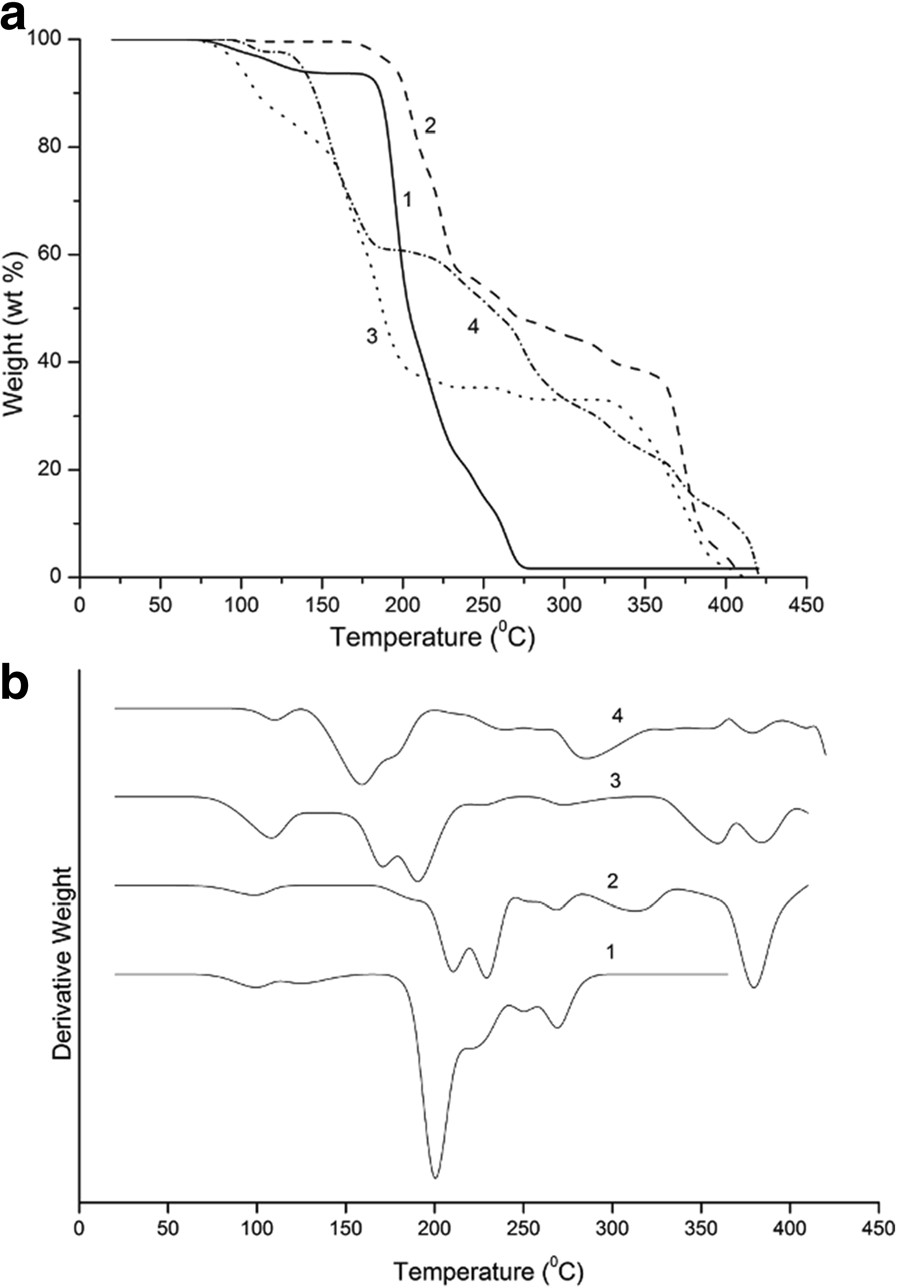
Thermogravimetric analysis **a** heating curves, and **b** their first derivatives for the initial cellulose (*1*) initial cellulose after grinding to 93 °SR (*2*), cellulose after grinding and 43 % sulfuric acid hydrolysis (*3*), and cellulose after additional sonication (*4*)

Thermal stability of cellulose samples using thermogravimetric analysis was assessed. The thermal gravimetric analysis and differential thermal gravimetric curves of air-dried samples of initial cellulose, initial cellulose after grinding up to 93 °SR, cellulose after grinding and 43 % sulfuric acid hydrolysis, and cellulose after additional sonication are shown in Fig. 7. Differential thermal gravimetric curves indicate that all types of cellulose exhibited a small peak at about 80–110 °C due to the evaporation of adsorbed water. The temperature at which the maximum weight loss was triggered was 200 °C for initial cellulose. Cellulose after grinding up to 93 °SR was thermally stable with the temperature at which the maximum weight loss at 220–240 °C. Grinded cellulose was more thermally stable due to the formation of strong hydrogen bonds between separate fibrillated cellulose fibers. The temperatures of the degradation of cellulose after grinding, 43 % sulfuric acid hydrolysis, and cellulose after additional sonication were 170 and 150 °C, respectively. All cellulose samples had final degradation temperature of about 400 °C. The degradation behavior of the cellulose that underwent sulfuric acid hydrolysis was different from that of the initial cellulose and showed a lower degradation temperature. The low degradation temperature of the mechanochemically treated cellulose was due to an increase in a number of free-end chains of the cellulose. These end chains started to decompose at the lower temperatures, as have been previously shown [32]. Our data also support previous findings that the sulfate groups, introduced during hydrolysis, can work as flame retardant in such a way they cause an increase in the char fraction [33].

## Conclusions

In the present study, the effect of the mechanical treatment, hydrolysis, and sonication of the bleached softwood sulfate pulp on the optical and mechanical properties of the transparent nanocellulose films was assessed. It was established that the method of subsequent grinding, acid hydrolysis, and ultrasound treatment of the cellulose produces films, which are characterized by the transparency in the visible spectral range (up to 78 % at 600 nm), high Young’s modulus (up to 8.8 GPa), and tensile strength (up to 88 MPa) due to the increase in the order of the packing of the cellulose macromolecules.
